# ClustENMD: efficient sampling of biomolecular conformational space at atomic resolution

**DOI:** 10.1093/bioinformatics/btab496

**Published:** 2021-07-08

**Authors:** Burak T Kaynak, She Zhang, Ivet Bahar, Pemra Doruker

**Affiliations:** Department of Computational and Systems Biology, School of Medicine, University of Pittsburgh, Pittsburgh, PA 15260, USA; Department of Computational and Systems Biology, School of Medicine, University of Pittsburgh, Pittsburgh, PA 15260, USA; Department of Computational and Systems Biology, School of Medicine, University of Pittsburgh, Pittsburgh, PA 15260, USA; Department of Computational and Systems Biology, School of Medicine, University of Pittsburgh, Pittsburgh, PA 15260, USA

## Abstract

**Summary:**

Efficient sampling of conformational space is essential for elucidating functional/allosteric mechanisms of proteins and generating ensembles of conformers for docking applications. However, unbiased sampling is still a challenge especially for highly flexible and/or large systems. To address this challenge, we describe a new implementation of our computationally efficient algorithm ClustENMD that is integrated with ProDy and OpenMM softwares. This hybrid method performs iterative cycles of conformer generation using elastic network model for deformations along global modes, followed by clustering and short molecular dynamics simulations. *ProDy* framework enables full automation and analysis of generated conformers and visualization of their distributions in the essential subspace.

**Availability and implementation:**

ClustENMD is open-source and freely available under MIT License from https://github.com/prody/ProDy.

**Supplementary information:**

Supplementary data are available at *Bioinformatics* online.

## 1 Introduction

Mapping the conformational space of proteins has been a challenge, especially for large assemblies of complexes. Elastic network models (ENMs) and normal mode analysis (NMA) have proven to predict the global modes of motion of biomolecular systems, and particularly supramolecular machines in the last two decades, as shown in numerous comparisons with experimentally observed conformational changes (Bahar *et al.*, 2010; Bakan and Bahar, 2009; Tama and Sanejouand, 2001). Thus, hybrid techniques combining ENM/NMA with molecular dynamics (MD) have come forth as computationally efficient means for elucidating transition pathways (Gur *et al.*, 2013; Orellana *et al.*, 2019) and for conformational sampling for large complexes (Costa *et al.*, 2015; Kurkcuoglu *et al.*, 2016), as we recently reviewed (Krieger *et al.*, 2020).

ClustENM hybrid algorithm (Kurkcuoglu *et al.*, 2016) has been introduced for *unbiased sampling* of the essential subspace spanned by the softest ENM modes through integration with clustering and energy minimization of conformers. Comparison with experimental data has shown the efficiency and utility of ClustENM for investigating highly flexible proteins like calmodulin (Kurkcuoglu and Doruker, 2016; Kurkcuoglu *et al.*, 2016) as well as large assemblies such as the ribosome (Can *et al.*, 2017; Guzel *et al.*, 2020; Kurkcuoglu *et al.*, 2016). More recently, ClustENM conformers have proven to facilitate protein–DNA and protein–protein ensemble docking (Kurkcuoglu and Bonvin, 2020) and prediction of cryptic allosteric pockets (Kaynak *et al.*, 2020).

Such promising results have motivated us to further develop and implement the ClustENMD version in the widely used *ProDy* (Bakan *et al.*, 2011; Zhang *et al.*, 2021) application programming interface (API) via integration with OpenMM (Eastman *et al.*, 2017) software. This version allows us to generate more realistic conformers by performing short MD simulations even for large allosteric complexes, together with high efficiency and full automation within a Python environment.

## 2 Methods and features

**Fig. 1.**
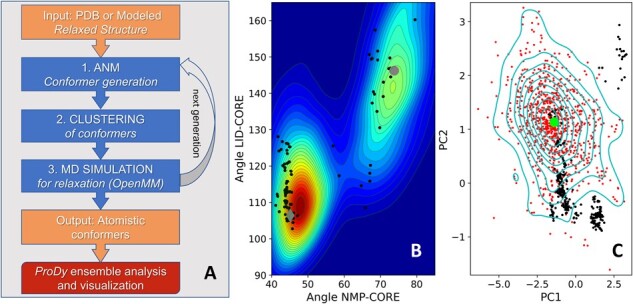
ClustENMD workflow and conformational sampling. (**A**) ClustENMD algorithm. (**B**) *AK*. Population distribution of ClustENMD conformers is plotted on the LID-Core and NMP-Core angles space of AK. This distribution contains a total of 1804 conformers, namely three runs starting from both open (*gray circle*) and closed (*gray diamond*) states. Experimental structures are depicted as black circles. (**C**) *HIV-1 RT*. Conformational surface plotted along the first two PCs of experimental structures (*black dots*), onto which conformers and the initial structure (*green circle*) are projected. *Cyan contours* indicate the density levels of 903 ClustENMD conformers. The distributions in panels B and C were produced by kernel density estimate (KDE) using Gaussian kernels.

The ClustENMD algorithm is explained schematically in Figure 1A. In *Step 1*, the input structure is subjected to anisotropic network model (ANM) analysis to produce atomistic conformers using random deformations along linear combinations of a set of global ENM modes. In *Step 2*, the conformers are clustered based on their structural similarities, and a representative member is selected for each cluster. In *Step 3*, the representatives from the previous step are structurally relaxed by short MD simulations using OpenMM. The new conformers are then fed back to *Step 1*, each being used as a starting point for a new generation of conformers. This iterative procedure (*Steps 1–3*) is repeated for several generations to allow for sufficiently large excursions from the original energy minimum.

ClustENMD has the following features (see Supplementary Material including Tutorial for details):

- Implemented as a class in *ProDy*
- Integrated with OpenMM (*Step 3*)
- Applicable to multimeric complexes/assemblies, comprising protein, RNA and/or DNA chains
- Input structure either retrieved from the Protein Data Bank (Berman *et al.*, 2000) (PDB) or provided by the user in PDB file format
- Addition of hydrogens and any missing heavy atoms in the residues of the input structure by PDBFixer/OpenMM
- MD simulations performed by OpenMM, either in implicit solvent (Onufriev *et al.*, 2004) [Amber99SB (Lindorff-Larsen *et al.*, 2010) forcefield] or explicit solvent [Amber14 (Maier *et al.*, 2015) with TIP3P-FB (Wang *et al.*, 2014) forcefields]
- ANM (Atilgan *et al.*, 2001) for conformer generation using a set of global modes (*ProDy*)
- Pairwise root-mean-square deviation (RMSD)-based hierarchical clustering
- Atomic coordinates of conformers saved as *ProDy* ensemble, and/or in PDB/DCD format
- Analysis of output conformers using diverse *ProDy* modules, e.g. ensemble analysis
- Fully automated pipeline, from input PDB file to the generated ensemble of conformers
- High-computational efficiency on GPU-architecture

## 3 Illustration

ClustENMD results for two case studies are presented in Figure 1 using simulations in implicit solvent model and heating up (HU) the system to 300 K. Figure 1B presents the population distribution for adenylate kinase (AK). AK is known to undergo a large conformational change (7 Å RMSD) between open (apo) and closed (substrate/inhibitor-bound) states. The contour plot corresponding to the population distribution of ClustENMD conformers is displayed on the two-dimensional (2D) space spanned by the two inter-domain angles, namely LID-Core and NMP-Core (Beckstein *et al.*, 2009). This plot is based on six independent runs, each comprising five generations (see Supplementary Table S1 for details on all systems/runs). Homologous experimental structures retrieved from the PDB using *ProDy* are shown on the same plot (*black dots*). ClustENMD conformers sample the two states as well as the transition region between them. See Supplementary Figure S1 for runs with more generations.

Figure 1C displays the 2D space for hetero-dimeric HIV-1 reverse transcriptase (RT), a large enzyme (*N* = 1000 residues). Here, the population distribution (contour plot) is projected onto the essential space of experimentally resolved structures. The axes denoted by the first two principal components (PC1 and PC2) are derived from the principal component analysis of 365 experimental structures (*black circles*) resolved for RT under different conditions (oligonucleotide/inhibitor-bound or -unbound). ClustENMD conformers (*red circles*) projected onto this space sample the close neighborhoods of most experimental structures (see Supplementary Fig. S2 for other runs including those in explicit solvent). We also present in Supplementary Figure S3, the counterpart of Figure 1C for AK, i.e. the generated conformers projected onto the subspaces spanned by experimentally defined PCs. Supplementary Figures S4 and S5 further display the respective ensemble of RT conformers in the space spanned by fingers-thumb versus fingers-RNase H distances, and that of HIV-1 protease conformers projected onto PC1–PC2 subspace.

The high efficiency of ClustENMD is reflected by the average computing time for a 5-generation run that generates 300 conformers (presented in Fig. 1), which takes 8 min (AK, 214 residues) to 27 min (RT, 978 residues) on a single GPU platform with NVIDIA^®^ GeForce^®^ RTX 2080 Ti graphics card. For a comparison of computational efficiency and required resources, we refer to a recent enhanced sampling study on generating a detailed free energy landscape for AK using Gaussian accelerated replica-exchange umbrella sampling (Oshima *et al.*, 2019); each of the 32 replica (of 2 × 10^8^ MD time steps) would require a couple of days on the same platform, as opposed to a total run time of 8 min for ClustENMD. We note that ClustENMD could be applied to the protein model refinement problem if the excursions/deformations from the initial model are restricted, possibly by adding restraints (Heo *et al.*, 2021), in order to retain the initial model’s conformational characteristics. It remains to be seen in future applications whether such an approach might help to efficiently sample conformations closer to the native structure.

## 4 Concluding remarks

The ClustENMD algorithm is implemented within *ProDy* (Bakan *et al.*, 2011; Zhang *et al.*, 2021), an open-source Python API for protein structure, dynamics and sequence analysis, containing multiple modules. The *ProDy* package (downloaded more than 2.1 million times and visited by 140 000+ unique users worldwide) ensures broad dissemination of ClustENMD to the research community in addition to providing accessory tools for analyses and visualization. The current version of ClustENMD is unique in performing unbiased sampling with high-computational efficiency, augmented by fully automated and user-friendly features upon integration with *ProDy* and OpenMM.

## Funding

This work has been supported by the National Institutes of Health [P41GM103712 to I.B.].

*Conflict of Interest*: none declared.

## Supplementary Material

btab496_Supplementary_Data

## References

[btab496-B1] Atilgan A.R. et al (2001) Anisotropy of fluctuation dynamics of proteins with an elastic network model. Biophys. J., 80, 505–515. 11159421 10.1016/S0006-3495(01)76033-X PMC1301252

[btab496-B2] Bahar I. et al (2010) Global dynamics of proteins: bridging between structure and function. Annu. Rev. Biophys., 39, 23–42. 20192781 10.1146/annurev.biophys.093008.131258 PMC2938190

[btab496-B3] Bakan A. , Bahar I. (2009) The intrinsic dynamics of enzymes plays a dominant role in determining the structural changes induced upon inhibitor binding. Proc. Natl. Acad. Sci. USA, 106, 14349–14354. 19706521 10.1073/pnas.0904214106 PMC2728110

[btab496-B4] Bakan A. et al (2011) ProDy: protein dynamics inferred from theory and experiments. Bioinformatics, 27, 1575–1577. 21471012 10.1093/bioinformatics/btr168 PMC3102222

[btab496-B5] Beckstein O. et al (2009) Zipping and unzipping of adenylate kinase: atomistic insights into the ensemble of open<–>closed transitions. J. Mol. Biol., 394, 160–176. 19751742 10.1016/j.jmb.2009.09.009 PMC2803350

[btab496-B6] Berman H.M. et al (2000) The Protein Data Bank. Nucleic Acids Res., 28, 235–242. 10592235 10.1093/nar/28.1.235 PMC102472

[btab496-B7] Can M.T. et al (2017) Conformational dynamics of bacterial trigger factor in apo and ribosome-bound states. PLoS One, 12, e0176262. 28437479 10.1371/journal.pone.0176262 PMC5402958

[btab496-B8] Costa M.G. et al (2015) Exploring free energy landscapes of large conformational changes: molecular dynamics with excited normal modes. J. Chem. Theory Comput., 11, 2755–2767. 26575568 10.1021/acs.jctc.5b00003

[btab496-B9] Eastman P. et al (2017) OpenMM 7: rapid development of high performance algorithms for molecular dynamics. PLoS Comput. Biol., 13, e1005659. 28746339 10.1371/journal.pcbi.1005659 PMC5549999

[btab496-B10] Gur M. et al (2013) Global transitions of proteins explored by a multiscale hybrid methodology: application to adenylate kinase. Biophys. J., 105, 1643–1652. 24094405 10.1016/j.bpj.2013.07.058 PMC3791301

[btab496-B11] Guzel P. et al (2020) Exploring allosteric signaling in the exit tunnel of the bacterial ribosome by molecular dynamics simulations and residue network model. Front. Mol. Biosci., 7, 586075. 33102529 10.3389/fmolb.2020.586075 PMC7545307

[btab496-B12] Heo L. et al (2021) Improved sampling strategies for protein model refinement based on molecular dynamics simulation. J. Chem. Theory Comput., 17, 1931–1943. 33562962 10.1021/acs.jctc.0c01238 PMC7946773

[btab496-B13] Kaynak B.T. et al (2020) Essential site scanning analysis: a new approach for detecting sites that modulate the dispersion of protein global motions. Comput. Struct. Biotechnol. J., 18, 1577–1586. 32637054 10.1016/j.csbj.2020.06.020 PMC7330491

[btab496-B14] Krieger J.M. et al (2020) Towards gaining sight of multiscale events: utilizing network models and normal modes in hybrid methods. Curr. Opin. Struct. Biol., 64, 34–41. 32622329 10.1016/j.sbi.2020.05.013 PMC7666066

[btab496-B15] Kurkcuoglu Z. , Bonvin A. (2020) Pre- and post-docking sampling of conformational changes using ClustENM and HADDOCK for protein-protein and protein-DNA systems. Proteins, 88, 292–306. 31441121 10.1002/prot.25802 PMC6973081

[btab496-B16] Kurkcuoglu Z. , Doruker P. (2016) Ligand docking to intermediate and close-to-bound conformers generated by an elastic network model based algorithm for highly flexible proteins. PLoS One, 11, e0158063. 27348230 10.1371/journal.pone.0158063 PMC4922591

[btab496-B17] Kurkcuoglu Z. et al (2016) ClustENM: ENM-based sampling of essential conformational space at full atomic resolution. J. Chem. Theory Comput., 12, 4549–4562. 27494296 10.1021/acs.jctc.6b00319 PMC5088496

[btab496-B18] Lindorff-Larsen K. et al (2010) Improved side-chain torsion potentials for the Amber ff99SB protein force field. Proteins, 78, 1950–1958. 20408171 10.1002/prot.22711 PMC2970904

[btab496-B19] Maier J.A. et al (2015) ff14SB: improving the accuracy of protein side chain and backbone parameters from ff99SB. J. Chem. Theory Comput., 11, 3696–3713. 26574453 10.1021/acs.jctc.5b00255 PMC4821407

[btab496-B20] Onufriev A. et al (2004) Exploring protein native states and large-scale conformational changes with a modified generalized born model. Proteins, 55, 383–394. 15048829 10.1002/prot.20033

[btab496-B21] Orellana L. et al (2019) eBDIMS server: protein transition pathways with ensemble analysis in 2D-motion spaces. Bioinformatics, 35, 3505–3507. 30838394 10.1093/bioinformatics/btz104 PMC6748756

[btab496-B22] Oshima H. et al (2019) Replica-exchange umbrella sampling combined with Gaussian accelerated molecular dynamics for free-energy calculation of biomolecules. J. Chem. Theory Comput., 15, 5199–5208. 31539245 10.1021/acs.jctc.9b00761

[btab496-B23] Tama F. , Sanejouand Y.H. (2001) Conformational change of proteins arising from normal mode calculations. Protein Eng., 14, 1–6. 11287673 10.1093/protein/14.1.1

[btab496-B24] Wang L.P. et al (2014) Building force fields: an automatic, systematic, and reproducible approach. J. Phys. Chem. Lett., 5, 1885–1891. 26273869 10.1021/jz500737m PMC9649520

[btab496-B25] Zhang S. et al (2021) ProDy 2.0: increased scale and scope after 10 years of protein dynamics modelling with Python. Bioinformatics, btab187. doi:10.1093/bioinformatics/btab187/6211036. 10.1093/bioinformatics/btab187 PMC8545336 33822884

